# Students' Emotional Well-being and Academic Functioning Before, During, and After Lockdown in Germany: Cohort Study

**DOI:** 10.2196/34388

**Published:** 2022-11-15

**Authors:** Tania R Nuñez, Nina Pallasch, Theda Radtke

**Affiliations:** 1 Department of Health Psychology and Applied Diagnostics Institute of Psychology University of Wuppertal Wuppertal Germany; 2 Department of Psychology Lund University Lund Sweden

**Keywords:** self-efficacy, academic self-concept, test anxiety, achievement motivation, positive and negative affect, mobile phone, COVID-19

## Abstract

**Background:**

The COVID-19 lockdowns have led to social detriments and altered learning environments among university students. Recent research indicates that such ramifications may engender various impairments to students’ mental health. However, such research has major limitations, such as the lack of a prepandemic control measure, the focus on singular well-being parameters, or the investigation of only the early phases of the pandemic.

**Objective:**

To address these research gaps, this comprehensive and nationwide study compared 3 student cohorts (aged 17-48 years) in Germany: a *prepandemic cohort* (January-February 2020), a *postlockdown cohort* (May 2020-July 2020), and an *intralockdown cohort* (January-February 2021) regarding students’ general emotional well-being and academic functioning. It was hypothesized that, because of rigorous lockdown-related restrictions, students in the intralockdown cohort would report diminished general emotional well-being compared with the other cohorts. Furthermore, because of ongoing remote learning since the beginning of the pandemic, it was expected that students’ academic functioning would decrease across all 3 cohorts.

**Methods:**

The data collection was performed over 3 consecutive semesters (fall semester 2019-2020, spring semester 2020, and fall semester 2020-2021). Students were surveyed on the web on various aspects regarding their general emotional well-being (eg, stress and general well-being) and academic functioning (eg, concentration and study-related flow). Data analyses were performed using multivariate ANOVAs.

**Results:**

A total of 787 students participated in this study. Results indicated higher general well-being in the postlockdown cohort than in the intralockdown cohort (*P*=.02). As for students’ academic functioning, our results revealed that students in the prepandemic cohort reported higher study-related flow (*P*=.007) and concentration (*P*=.001) than those in the intralockdown cohort. In addition, students reported higher flow (*P*=.04) and concentration (*P*=.04) in the postlockdown cohort than those in the intralockdown cohort. No cohort effects were revealed for other aspects of general emotional well-being (eg, perceived stress) and academic functioning (eg, procrastination).

**Conclusions:**

This study indicates that students’ general emotional well-being as well as motivational and attentional components of academic functioning can be impaired owing to the COVID-19 lockdowns and ongoing remote learning formats. The necessity and design of interventional programs remedying such effects in light of the ongoing crisis need to be addressed.

## Introduction

### Background

The novel COVID-19 outbreak, which was declared a global pandemic on March 11, 2020, has altered people’s everyday lives in an unparalleled way. With a total of 240,940,937 confirmed cases and 4,903,911 COVID-19–related deaths worldwide reported by the *World Health Organization* [1] at the time of writing this paper, it becomes abundantly clear that the disease is a serious threat to people’s physical health [2-4]. However, research on large-scale health crises and quarantine-like situations [5] as well as recent work concerning COVID-19 [6-8] suggest that the pandemic and concomitant restrictions may have led to a multitude of ramifications similarly affecting people’s mental health.

For university students, such ramifications have been manifold. Nationwide lockdowns have not only led to incisive limitations regarding general social contact and peer relationships but also to altered learning environments because of a rapid shift from in-person to remote learning [9]. Although coinciding with multiple other lockdown-related stressors (eg, student job loss, insecurity about mandatory internships and future employment, and fears about contracting the disease) [9,10], such social and academic ramifications can engender ample mental health detriments affecting students’ both general emotional well-being [7,8,11,12] and academic functioning [13-15].

### Students’ Emotional Well-being

Cross-sectional studies during the early phases of the pandemic suggest that COVID-19 lockdowns have led to a high prevalence of sadness and frustration [16], depressive symptoms [17-19], anxiety and stress [17,20-26], and sleep disturbances [16] among students. Furthermore, adverse changes in such lockdown-related mental health aspects have been found in studies using both retrospective [27] as well as actual prepandemic control measures [7,8,12]. For instance, a study comparing 3 student cohorts tested in fall 2019 (prepandemic measure), spring 2020 (when lockdown provisions were initiated), and fall 2020 (when lockdown provisions were eased) revealed increases in depression, anger, stress, and mania between the fall 2019 cohort and the spring 2020 cohort [7]. However, results showed no differences between the prepandemic and the fall 2020 cohort. Another study comparing students’ depressive symptoms before the lockdown in Italy (October 2019 and December 2019), during the lockdown (April 2020), and after the lockdown was lifted (May 2020 and June 2020) showed similar results [8].

In summary, research indicates that COVID-19 lockdowns may have an adverse effect on students’ general emotional well-being. However, studies have also shown that students appear to recover quickly from well-being–related detriments once lockdown provisions are eased [7] or lifted [8]. Still, it should be noted that, despite the possibility of rapid recovery, severe periodic emotional impairments can come with long-term consequences for students, who have been shown across studies to be a vulnerable group regarding mental health problems [12,28-30]. Furthermore, such impairments may also create momentary as well as future costs regarding academic functioning [13,14,31]. Consequently, more research is needed to gain comprehensive insights into lockdown-related and possible long-term consequences.

### Students’ Academic Functioning

Stressful situations coupled with social isolation and web-based learning can similarly compromise not only general emotional well-being but also academic functioning. On the one hand, web-based teaching during the COVID-19 pandemic had to be implemented rapidly, which may have led to a lack in adequate design and organization of web-based teaching formats as well as uncertainty and anxiety regarding course work and exam preparations [32]. Such shortcomings can result in heightened self-doubt and difficulties in information processing on the part of the students [9,20,25,33]. By contrast, research has shown that because of COVID-19 lockdown provisions, people have vastly increased their overall technology use [34]. Although technology-mediated communication has been an important way of staying socially and academically connected during the COVID-19 lockdowns [35], increased screen time and the simultaneous use of technologies for different everyday tasks (eg, staying connected with friends and family, schoolwork, and entertainment) can engender cognitive (eg, inability to concentrate) and performance-related (eg, worse grades) detriments [36,37].

Research on the effects of COVID-19 lockdowns and remote learning arrangements on students’ academic functioning is limited. However, qualitative data derived from interviews conducted with students in the United States showed that 81.5% (159/195) of interviewees stated concerns about their general academic performance because of the COVID-19 pandemic [25]. Furthermore, 88.7% (173/195) of interviewees indicated that they were negatively affected in their ability to concentrate on schoolwork. Other qualitative [38] and quantitative yet observational studies [23,33] corroborated these findings. Other studies revealed that students, compared with a retrospectively assessed control measure, reported declines in their motivation [14,31] as well as their behavioral and emotional academic engagement [31]. Moreover, it has been found that students experienced decreased attention and heightened externalizing problems [39] as well as increased study-related stress during the early phase of the pandemic compared with the time before [13].

In summary, lockdown-related social detriments as well as (emergency) remote learning may have taken a toll on students’ academic functioning, including factors such as motivation, concentration, and study-related stress. However, existing studies using prepandemic control measures are extremely scarce. Thus, more research is needed to gain a deeper insight into the changes in students’ academic functioning in the context of COVID-19 lockdowns and investigate which aspects of academic functioning may be particularly affected by the crisis.

### Aims of This Study

To develop interventional programs remedying emotional and academic complications among the vulnerable population of students [12,28-30], more research on the effects of the COVID-19 lockdowns on the student population is needed. However, recent research has major limitations. First, only few studies incorporate a control measure (ie, a prepandemic measure) into their investigations [7,8,39]. Second, many studies are limited to 1 university [7,13,40] or 1 specific study department of a singular institution [12], limiting the generalizability of the results obtained. Third, most studies have investigated the effects of the COVID-19 crisis on students during its early onset but not in later phases of the pandemic [8,12-14]. Fourth, recent studies have mainly focused on few student characteristics such as stress [13,27,41], depression [42], or acceptance of web-based formats [43], thus neglecting a comprehensive examination of students’ well-being.

**Figure 1:**
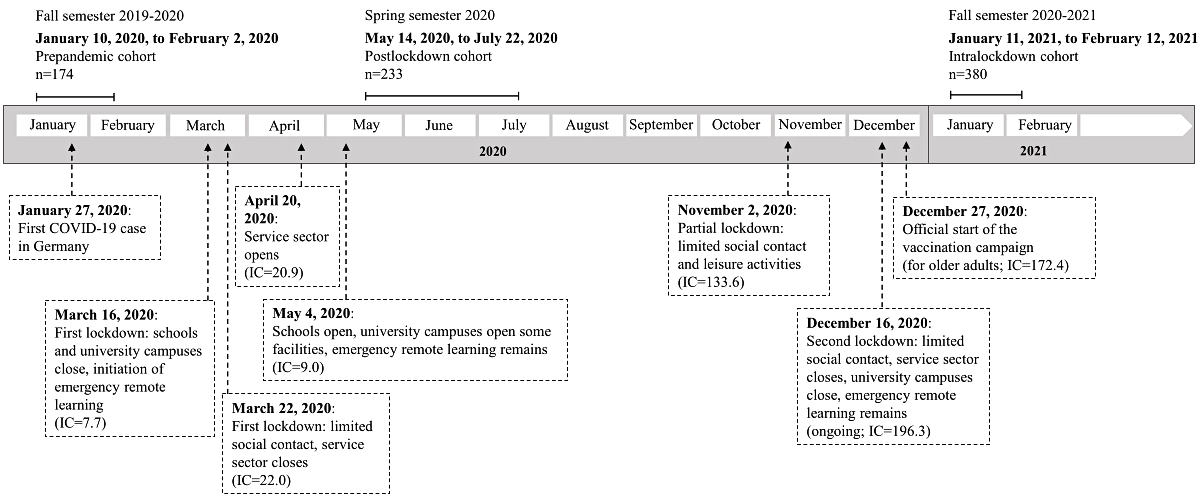
Visualization of the data collection process in relation to the most relevant pandemic-related events in Germany between January 2020 and February 2021, including incidence values. IC: new COVID-19 infections during the last 7 days per inhabitant×100,000.

To address these research gaps, this study aimed to compare between-subject data gathered in a comprehensive research project on students’ mental health and academic functioning encompassing 3 student cohorts: a *prepandemic cohort* (ie, a student cohort tested before the pandemic), a *postlockdown cohort* (ie, a student cohort tested after the first lockdown in Germany, when social and everyday life restrictions were eased but remote teaching at universities continued), and an *intralockdown cohort* (ie, a student cohort tested during the second lockdown in Germany, when social and everyday life restrictions were reinstated in addition to remote teaching and web-based examinations; Figure 1).

### Hypotheses

#### Students’ General Emotional Well-being

Research indicates that general social detriments [44] and COVID-19–related lockdown provisions [19,22,45] can engender affective detriments in students (eg, negative emotions, stress, and depression). However, research also shows that students’ general emotional well-being appears to rapidly improve to prepandemic levels when lockdown-related restrictions are eased [7] or lifted [8]. In line with this, for students’ general emotional well-being, hypothesis 1 is given in Textbox 1.

**Hypothesis 1.**

**Hypothesis 1.1**

- The intralockdown cohort will report (a) less positive and (b) more negative affect, (c) less general well-being, and (d) higher perceived stress than the prepandemic cohort.

**Hypothesis 1.2**

- The intralockdown cohort will report (a) less positive and (b) more negative affect, (c) less general well-being, and (d) higher perceived stress than the postlockdown cohort.

**Hypothesis 1.3**

- The postlockdown cohort will report similar levels of (a) positive and (b) negative affect, (c) general well-being, and (d) perceived stress compared with the prepandemic cohort.

#### Students’ Academic Functioning

Research also indicates that general social deprivation [15] and COVID-19–related lockdowns [14,23,33] can impair students’ academic functioning [46,47]. Therefore, we assume that the intralockdown cohort will exhibit a severe decline in their academic functioning compared with the prepandemic cohort. However, the postlockdown cohort should also be affected in their academic functioning owing to remaining emergency remote learning, albeit not as pronounced as those students assessed during the second lockdown.

Therefore, as for students’ academic functioning encompassing study-related emotional well-being (hypotheses 2a-2b), academic self-perception (hypotheses 2c-2d), motivation (hypotheses 2e-2f), and self-regulation (hypotheses 2g-2i), we have given hypothesis 2 in Textbox 2.

**Hypothesis 2.**

**Hypothesis 2.1**

- The intralockdown cohort will report (a) more study-related stress, (b) more test anxiety, (c) a lower academic self-concept, (d) less study-related self-efficacy, (e) adverse achievement motivation, (f) less study-related flow, (g) less concentration, (h) lower frequency of study activities, and (i) more procrastination than the prepandemic cohort.

**Hypothesis 2.2**

- The intralockdown cohort will report (a) more study-related stress, (b) more test anxiety, (c) a lower academic self-concept, (d) less study-related self-efficacy, (e) adverse achievement motivation, (f) less study-related flow, (g) less concentration, (h) lower frequency of study activities, and (i) more procrastination than the postlockdown cohort.

**Hypothesis 2.3**

- The postlockdown cohort will report (a) more study-related stress, (b) more test anxiety, (c) a lower academic self-concept, (d) less study-related self-efficacy, (e) adverse achievement motivation, (f) less study-related flow, (g) less concentration, (h) lower frequency of study activities, and (i) more procrastination than the prepandemic cohort.

## Methods

### Study Design

This study is part of a large longitudinal randomized controlled study investigating the effectiveness of a planning intervention of reduced smartphone interference on students’ mental health. In the longitudinal study, 3 student cohorts were investigated on various mental health aspects as well as smartphone use behaviors over 5 time points. In this study, preinterventional baseline data related to self-reported general emotional well-being and academic functioning from all 3 student cohorts (ie, prepandemic cohort, postlockdown cohort, and intralockdown cohort) were used (trial registration: ClinicalTrials.gov NCT04550286).

### Recruitment

Participants were recruited nationwide through on- (only for the prepandemic cohort) and off-campus advertisements, social media platforms, and universities’ listservs. The inclusion criteria for the study were participants who were students, were aged ≥16 years, had at least one graded examination during the exam period of the respective semester, possessed sound knowledge of the German language, and used an Android smartphone regularly. Individuals who failed to meet these inclusion criteria were excluded from study participation (Multimedia Appendix 1).

### Procedure

As illustrated in Figure 1, between-subject data were collected via the platform *SoSci Survey* (SoSci Survey GmbH) in 3 consecutive semesters. The prepandemic cohort was tested between January 10, 2020, and February 2, 2020; the postlockdown cohort was tested between May 14, 2020, and July 22, 2020; and the intralockdown cohort was tested between January 11, 2021, and February 12, 2021. During the first 2 lockdowns that were instated in Germany, the population faced vast restrictions regarding social contact and the closure of non–system-relevant service sector industries as well as primary, secondary, and tertiary institutions of education. Restaurants, cafés, clubs, and other gastronomic and entertainment establishments were closed. Sports centers had to discontinue classes and other activities. Parks were locked or limited to a certain number of individuals, and pedestrians were not allowed to remain in larger groups. Visits to hospitals and homes for older adults were prohibited. Many began to work remotely, and schools as well university campuses were shut down. As a consequence, many universities transitioned from in-person to emergency remote learning. In May 2020, restrictions related to the first lockdown were eased, allowing for small social gatherings and the opening of the service sector. However, although universities alleviated access limitations to campuses and allowed for some in-person examinations under rigorous hygiene concepts, they continued with remote teaching formats. Remote teaching was continued during the second lockdown (Figure 1).

Participation in this study was voluntary; students of the institution responsible for the study’s conduction received course credit. All participants were given the opportunity to take part in a raffle of various prizes, including adventure activity gift cards and other vouchers (worth US $830).

### Ethics Approval

Participants had to give their informed consent to take part in the study and were treated in accordance with the ethical standards of the Helsinki Declaration [48]. The study was approved by the Witten and Herdecke University Ethics Commission (215/2019).

### Measures

#### General Emotional Well-being

##### Positive and Negative Affect

Students’ positive and negative affect was investigated using a shortened German version of the *Positive and Negative Affect Schedule* [49,50]. Participants were to indicate how they had felt during the last 7 days on 5 items covering positive emotions (eg, “attentive”; ω=0.77) and 5 items covering negative emotions and emotional expressions (eg, “upset”; ω=0.80). Responses were to be given on a 5-point Likert scale ranging from 1=*not at all* to 5=*extremely*.

##### General Well-being

Students’ general well-being was measured using a German version of the *World Health Organization-5 Well-being Index* [51]. Regarding the last 7 days, participants were to appraise their well-being on 5 items (eg, “I felt calm and relaxed”; ω=0.83) on a 6-point Likert scale ranging from 0=*never* to 5=*all the time*. A well-being index score of 13 represents low well-being and can be used as a screening marker for the presence of depressive symptoms.

##### Perceived Stress

Perceived stress was measured using a German version of the *Perceived Stress Scale* [52,53]. Participants were to indicate how often they had perceived stress on 9 items (eg, “How often during the last 7 days...have you felt nervous and stressed”; ω=0.85) on a 5-point Likert scale ranging from 1=*never* to 5=*very often*.

#### Academic Functioning

##### Study-Related Stress

Students’ study-related stress was investigated using an adaptation of the stress measure used by Schmidt et al [54]. This measure consists of 4 items (eg, “During the last 7 days...I felt nervous and stressed due to the preparations for my exam(s)”; ω=0.88) that were to be answered on a 5-point Likert scale ranging from 1=*not at all* to 5=*extremely*.

##### Test Anxiety

Test anxiety was assessed using items adapted from the German version of the *Test Anxiety Inventory* [55,56]. The measure used consists of 10 items; 5 items address *test-related agitation* (*Test Anxiety Inventory-Emotionality*; eg, “My heart is in my mouth”; ω=0.89), whereas the other 5 items assess *test-related worries* (*Test Anxiety Inventory-Worry*; eg, “I wonder whether my performance will suffice”; ω=0.83). Participants were to respond on a 6-point Likert scale ranging from 1=*completely disagree* to 6=*completely agree*.

##### Academic Self-concept

Students’ academic self-concept was assessed using the subscale *academic self-concept* of the *German Scales regarding Academic Self-Concept* [57]. Academic self-concept encompasses students’ general perceptions and beliefs about their own academic capabilities [58]. The subscale used consists of 5 items (eg, “I think I am very intelligent”; ω=0.88) that were to be answered on a 7-point Likert scale ranging from 1=*completely disagree* to 7=*completely agree*.

##### Study-Related Self-efficacy

Students’ study-related self-efficacy was measured using the *German Self-efficacy Scale* [56], adapted to the student context. Study-related self-efficacy encompasses students’ expectations regarding possible achievements in a given context, which is thought to be a precursor of students’ academic self-concept [58]. This instrument comprises 7 items (eg, “During the last 7 days...I was sure that I can solve even the difficult tasks and texts for the exam if I make an effort”; ω=0.86). Participants were to respond on a 6-point Likert scale ranging from 1=*completely disagree* to 6=*completely agree*.

##### Achievement Motivation

Students’ achievement motivation was assessed using the *German Questionnaire for the Assessment of Current Motivation in Learning and Performance Situations* [59]. In this study, the subscales *probability of success* (4 items; eg, “I think everybody can pass this exam”; ω=0.81) and *probability of failure* (4 items; eg, “When thinking about the upcoming exam, I am somewhat worried”; ω=0.79) were used. Probability of success encompasses students’ expectations regarding the probability of performing well in an upcoming exam. Probability of failure, by contrast, encompasses students’ expectations regarding the probability of not being able to deal with exam pressures and, consequently, performing badly. Participants were to indicate their achievement motivation regarding the upcoming exams and their study engagement on a 5-point Likert scale ranging from 1=*completely disagree* to 5=*completely agree*.

##### Study-Related Flow

Study-related flow was assessed using the *German Measure for Flow Experience* [60]. Flow experience can be understood as an action-related (as opposed to target-related) motivator in the academic context. The flow measure used includes 13 items (eg, “During the last 7 days...I was completely absorbed in what I was studying”; ω=0.82). Participants were to respond on a 5-point Likert scale ranging from 1=*completely disagree* to 5=*completely agree*.

##### Concentration

Students’ concentration while studying was measured using the subscale *concentration* of the *German Study-Related Learning Strategy Scales* [61], which consists of 6 items (eg, “During the last 7 days...I was unconcentrated”; ω=0.94). Participants were to respond on a 5-point Likert scale ranging from 1=*completely disagree* to 5=*completely agree*.

##### Frequency of Study Activities

Students’ frequency of study activities was assessed using a self-developed measure. Students were to indicate on how many days during the last 7 days they had studied, at least for a little while (eg, memorizing information, reading, and doing exercises). Participants were to choose whether they had studied on *1 day*, *2 days*, *3 days*, *4 days*, *5 days*, *6 days*, *7 days*, or *no days*.

##### Procrastination

Students’ tendency to procrastinate was assessed using the German version of the *Aitken Procrastination Scale* [62]. Procrastination encompasses the tendency to postpone tasks, which may affect a broad range of activities and is generally independent of specific situational stimuli. This measure encompasses 13 items (eg, “I often need a long time to get things started”; ω=0.91) that participants were to answer on a 5-point Likert scale ranging from 1=*completely disagree* to 5=*completely agree*.

### Statistical Analysis

All data analyses were performed with SPSS Statistics (version 27; IBM Corp). Across variables, <1% of missing data (item nonresponses) were identified; thus, analyses were performed with the available information. To test our hypotheses, we performed multivariate analysis of variances (MANOVAs). For that, all variables assessed as indicators of general emotional well-being and all variables of the respective aspects regarding academic functioning (ie, study-related well-being, academic self-perception, academic motivation, and academic self-regulation; cf Measures) were conjointly analyzed.

Univariate outliers were identified using visual inspection of box plots and analyses of *z* scores. Multivariate outliers were identified using visual inspection of *Q*-*Q* plots and analyses of Mahalanobis distance. We identified 5 univariate outliers (|*z*|=3.29) and 2 multivariate outliers as assessed based on critical chi-square values (*P*<.001). Univariate outliers were winsorized. Multivariate outliers were not omitted as analyses yielded similar results whether extreme cases were excluded or not. Normality of outcomes was confirmed as assessed by visual inspection of histograms and Normal *Q*-*Q* Plots. Homogeneity of variances was tested using the Levene test, showing that, for most variables, variances were equal for all student cohorts, with significance values ranging from *P*=.05 (positive affect) to *P*=.99 (negative affect). The Levene test showed significant results only for study-related stress (*P*=.02). For multivariate analyses, homogeneity of covariance matrices was confirmed by the Box test (*P*>.05). Visual inspection of Normal *P*-*P* Plots and histograms plotting standardized predicted values against standardized residuals confirmed linearity and homoscedasticity. Furthermore, we detected no evidence of multicollinearity as assessed by the Pearson correlation (|*r*|<0.9) and the variance inflation index (variance inflation factor <2).

As recent COVID-19 studies have shown that demographic factors may influence general emotional well-being and academic functioning [6,17,22,26], we performed additional hypotheses tests including control variables (ie, gender, age, semester, and number of exams in the respective semester; Multimedia Appendices 2-6).

## Results

### Participants

A total of 787 participants were recruited for this study. The final sample of the prepandemic cohort consisted of 174 students (n=127, 73% women; n=46, 26.4% men; and n=1, 0.6% nonbinary) with a mean age of 22.89 (SD 3.58) years ranging from 17 to 38 years. The postlockdown cohort consisted of 233 students (n=172, 73.8% women; n=60, 25.8% men; and n=1, 0.4% nonbinary) with a mean age of 23.32 (SD 4.40) years ranging from 18 to 48 years. The intralockdown cohort encompassed 380 students (n=270, 71.1% women; n=106, 27.9% men; and n=4, 1.1% nonbinary) with a mean age of 22.47 (SD 3.31) years ranging from 17 to 43 years. Participants in the postlockdown cohort were, on average, slightly older than those in the other 2 cohorts (*F*_2,784_=3.80; *P*=.02; *η*_p_^2^=0.010). The distribution of men and women was equal across the 3 cohorts (N=781, *χ*^2^_2_=0.4; *P*=.81). The cohorts consisted of students from >200 German universities from all 16 federal states. Most participants were enrolled in higher semesters (579/787, 73.6%). First-year students were unequally distributed across cohorts, with relatively fewer first-year students in the postlockdown cohort (22/233, 9.4%) than in the prepandemic (53/174, 30.5%) and intralockdown (133/389, 35%) cohorts (N=787, *χ*^2^_2_=50.4; *P*<.001). Participants were enrolled in different study programs: arts and design (16/787, 2%), education studies (21/787, 2.7%), agricultural studies (25/787, 3.2%), cultural and language studies (29/787, 3.7%), psychology (62/787, 7.9%), social sciences (83/787, 10.5%), medicine and health (104/787, 13.2%), economy and law (139/787, 17.7%), natural sciences (140/787, 17.8%), and technology (165/787, 21%). On average, participants prepared for 4 (SD 1.51) exams during the respective semesters. The exam count was equal across cohorts (*F*_2,784_=2.89; *P*=.28; *η*_p_^2^=0.003). An overview of the sample demographics is provided in Table 1.

For the longitudinal study, the sample size was calculated a priori using G*Power [63]. The necessary sample size for identifying a small effect (Cohen *d*=0.17) regarding the smartphone interference reduction intervention in relation to a control group with a power of 1–*β*=.95 at a *P* value of .05 was 116. For this study, a post hoc power analysis was performed using G*Power. The analysis for the sample size of 787 resulted in a power of 1–*β*=.91 given a small cohort effect of *f*=0.13 and an error probability of *α*=.05.

**Table 1:** Demographics regarding all student cohorts (N=787).

			Prepandemic cohort (n=174)		Postlockdown cohort (n=233)		Intralockdown cohort (n=380)		Total
**Gender, n (%)**									
	Men	46 (26.4)		60 (25.8)		106 (27.9)		212 (26.9)	
	Women	127 (72.9)		172 (73.8)		270 (71.1)		569 (72.3)	
	Nonbinary	1 (0.6)		1 (0.4)		4 (1.1)		6 (0.8)	
**Semester information, n (%)**									
	First study	136 (78.2)		184 (79)		291 (76.6)		611 (77.6)	
	Second study	38 (21.8)		49 (21)		89 (23.4)		176 (22.4)	
	First-semester students	53 (30.5)		22 (9.4)		133 (35)		208 (26.4)	
	Higher-semester students	121 (69.5)		211 (90.6)		247 (65)		579 (73.6)	
**Study program, n (%)**									
	Arts and design	2 (1.1)		4 (1.7)		10 (2.6)		16 (2)	
	Education studies	4 (2.3)		7 (3)		10 (2.6)		21 (2.7)	
	Agricultural studies	4 (2.3)		4 (1.7)		17 (4.5)		25 (3.2)	
	Cultural and language studies	9 (5.2)		7 (3)		13 (3.4)		29 (3.7)	
	Psychology	27 (15.5)		12 (5.2)		23 (6.1)		62 (7.9)	
	Social sciences	11 (6.3)		31 (13.3)		41 (10.8)		83 (10.5)	
	Medicine and health	28 (16.1)		30 (12.9)		46 (12.1)		104 (13.2)	
	Economy and law	35 (20.1)		42 (18)		62 (16.3)		139 (17.7)	
	Natural sciences	21 (12.1)		43 (18.5)		76 (20)		140 (17.8)	
	Technology	31 (17.8)		53 (22.7)		81 (21.3)		165 (21)	
	Other	1 (0.6)		0 (0)		1 (0.3)		1 (0.1)	
**Federal state, n (%)**									
	Baden-Württemberg	38 (21.8)		40 (17.2)		54 (14.2)		132 (16.8)	
	Bavaria	13 (7.5)		58 (24.9)		80 (21.1)		151 (19.2)	
	Berlin	10 (5.7)		7 (3)		14 (3.7)		31 (3.9)	
	Brandenburg	0 (0)		11 (4.7)		5 (1.3)		16 (2)	
	Bremen	0 (0)		12 (5.2)		8 (2.1)		20 (2.5)	
	Hamburg	3 (1.7)		6 (2.6)		0 (0)		9 (1.1)	
	Hesse	17 (9.8)		8 (3.4)		57 (15)		82 (10.4)	
	Lower Saxony	3 (1.7)		8 (3.4)		33 (8.7)		44 (5.6)	
	Mecklenburg-Western Pomerania	14 (8)		24 (10.3)		4 (1.1)		42 (5.3)	
	North Rhine-Westphalia	40 (23)		4 (1.7)		67 (17.6)		111 (14.1)	
	Rhineland-Palatinate	4 (2.3)		17 (7.3)		6 (1.6)		27 (3.4)	
	Saarland	0 (0)		1 (0.4)		0 (0)		1 (0.1)	
	Saxony	8 (4.6)		18 (7.7)		10 (2.6)		36 (4.6)	
	Saxony-Anhalt	9 (5.2)		8 (3.4)		14 (3.7)		31 (3.9)	
	Schleswig-Holstein	0 (0)		5 (2.1)		4 (1.1)		9 (1.1)	
	Thuringia	14 (8)		6 (2.6)		24 (6.3)		44 (5.6)	
Age (years), mean (SD)			22.89 (3.58)		23.32 (4.40)		22.47 (3.31)		22.82 (3.73)
Exam count, mean (SD)			3.99 (1.57)		4.23 (1.56)		4.17 (1.45)		4.15 (1.51)

### Descriptive Outcome Analysis

Table 2 shows the mean values and SDs of all outcome variables for each student cohort. As for general emotional well-being, the descriptive data show that students reported positive and negative affect close to the average of the scale, indicating a moderate level. Students’ general well-being scores were below the cutoff value of 13, indicating low well-being in all student cohorts. Students in all cohorts also reported a perceived level of stress above the scale average (range 9-45). In line with this, students reported high study-related stress and increased worries regarding their upcoming exams (mean values were above the scale average). As for students’ self-perception, the data revealed scores above the average of the scale regarding academic self-concept and study-related self-efficacy. Furthermore, students reported high achievement motivation (mean values were above the scale average) with regard to their perceived probability of both success and failure. However, study-related flow was only moderate (mean values were close to the scale average). Similarly, students’ ability to concentrate was moderate, whereas the time that students reportedly engaged in their exam preparations was rather high (ie, >4.5 days per week on average). Finally, students’ tendency to procrastinate was also above the average of the scale.

Almost all outcome variables were correlated with one another, indicating that general emotional well-being and the different components of academic functioning were interconnected (see Table 3 for Pearson correlations of all outcome variables across cohorts). Noticeably, frequency of study activities was positively correlated only with positive affect (*r*_787_=0.18; *P*<.001), study-related flow (*r*_787_=0.15; *P*<.001), and concentration (*r*_787_=0.15; *P*<.001). All other outcome variables were not correlated with students’ study engagement.

**Table 2:** Descriptive data of outcomes, including mean scores, score range, and the effect size (*η*_p_^2^) for the differences among all cohorts.

		Prepandemic cohort, mean (SD)	Postlockdown cohort, mean (SD)	Intralockdown cohort, mean (SD)	Range	*η*_p_^2^
**General emotional well-being**						
	Positive affect	15.22 (3.75)	15.21 (3.14)	14.64 (3.53)	5-25	0.007
	Negative affect	10.83 (4.05)	10.88 (4.22)	11.23 (4.10)	5-25	0.002
	General well-being	11.51 (4.92)	11.89 (4.67)	10.92 (4.45)	0-25	0.008
	Perceived stress	25.56 (6.44)	26.57 (6.40)	26.88 (6.30)	9-45	0.007
**Study-related emotional well-being**						
	Study-related stress	3.12 (1.15)	3.14 (1.06)	3.14 (1.03)	1-4	<0.001
	Test anxiety (agitation)	3.34 (1.28)	3.25 (1.22)	3.36 (1.25)	1-6	0.001
	Test anxiety (worry)	4.46 (1.04)	4.40 (0.99)	4.40 (1.06)	1-6	<0.001
**Self-perception**						
	Academic self-concept	4.76 (1.03)	4.77 (0.91)	4.62 (0.98)	1-7	0.006
	Study-related self-efficacy	4.06 (0.76)	4.05 (0.79)	3.95 (0.88)	1-6	0.004
**Motivation**						
	Motivation (success)	3.51 (0.80)	3.48 (0.82)	3.40 (0.85)	1-5	0.003
	Motivation (failure)	3.32 (1.01)	3.18 (0.90)	3.35 (0.94)	1-5	0.007
	Study-related flow	2.69 (0.60)	2.65 (0.53)	2.55 (0.54)	1-5	0.012
**Self-regulation**						
	Concentration	2.54 (0.97)	2.42 (0.90)	2.25 (0.92)	1-5	0.017
	Frequency of study activities	4.64 (1.96)	4.89 (1.93)	4.83 (2.01)	0-7	0.002
	Procrastination	3.05 (0.81)	2.99 (0.83)	3.12 (0.78)	1-5	0.005

**Table 3:** Pearson correlations of outcome variables across cohorts.

	1	2	3	4	5	6	7	8	9	10	11	12	13	14	15
1. Positive affect	1	—^a^	—	—	—	—	—	—	—	—	—	—	—	—	—
2. Negative affect	−0.38^b^	1	—	—	—	—	—	—	—	—	—	—	—	—	—
3. General well-being	0.68^b^	−0.53^b^	1	—	—	—	—	—	—	—	—	—	—	—	—
4. Perceived stress	−0.51^b^	0.69^b^	−0.60^b^	1	—	—	—	—	—	—	—	—	—	—	—
5. Study-related stress	−0.32^b^	−0.58^b^	−0.50^b^	0.66^b^	1	—	—	—	—	—	—	—	—	—	—
6. Test anxiety (agitation)	−0.31^b^	0.67^b^	−0.43^b^	0.63^b^	0.66^b^	1	—	—	—	—	—	—	—	—	—
7. Test anxiety (worry)	−0.24^b^	0.52^b^	−0.37^b^	0.55^b^	0.59^b^	0.66^b^	1	—	—	—	—	—	—	—	—
8. Academic self-concept	0.39^b^	−0.31^b^	0.34^b^	−0.43^b^	−0.34^b^	−0.30^b^	−0.28^b^	1	—	—	—	—	—	—	—
9. Study-related self-efficacy	−0.43^b^	−0.39^b^	0.43^b^	−0.54^b^	−0.49^b^	−0.40^b^	−0.43^b^	0.64^b^	1	—	—	—	—	—	—
10. Achievement motivation (success)	0.34^b^	−0.40^b^	0.37^b^	−0.52^b^	−0.52^b^	−0.48^b^	−0.50^b^	0.50^b^	0.66^b^	1	—	—	—	—	—
11. Achievement motivation (failure)	−0.28^b^	0.52^b^	−0.39^b^	0.51^b^	0.50^b^	0.59^b^	0.67^b^	−0.24^b^	−0.37^b^	−0.40^b^	1	—	—	—	—
12. Study-related flow	0.55^b^	−0.29^b^	0.44^b^	−0.45^b^	−0.31^b^	−0.27^b^	−0.27^b^	0.36^b^	0.41^b^	0.37^b^	−0.28^b^	1	—	—	—
13. Concentration	0.42^b^	−0.36^b^	−0.34^b^	−0.40^b^	−0.32^b^	−0.34^b^	−0.32^b^	0.24^b^	0.23^b^	0.25^b^	−0.34^b^	0.58^b^	1	—	—
14. Frequency of study activities	0.18^b^	−0.012	0.029	−0.023	0.065	−0.010	0.036	0.028	0.001	0.033	−0.012	0.15^b^	0.15^b^	1	—
15. Procrastination	−0.43^b^	0.31^b^	−0.35^b^	0.40^b^	−0.33^b^	0.30^b^	0.25^b^	−0.27^b^	−0.26^b^	−0.29^b^	0.27^b^	−0.44^b^	−0.45^b^	−0.30^b^	1

^a^Not applicable.

^b^*P*<.001.

### Primary Outcome Analysis

#### General Emotional Well-being

MANOVA results for positive and negative affect, general well-being, and perceived stress with student cohort as independent factor indicated differences among the prepandemic, postlockdown, and intralockdown cohorts (*V*=0.020; *F*_8,1562_=2.00; *P*=.04; *η*_p_^2^=0.010). Univariate analyses indicated that the student cohort had an effect on general well-being (*F*_2,783_=3.32; *P*=.04; *η*_p_^2^=0.008) but neither on positive affect (*F*_2,783_=2.64; *P*=.07; *η*_p_^2^=0.007), negative affect (*F*_2,783_=0.81; *P*=.45; *η*_p_^2^=0.002), nor perceived stress (*F*_2,783_=2.63; *P*=.07; *η*_p_^2^=0.007).

Bonferroni-corrected post hoc comparisons for general well-being demonstrated that, although students in the intralockdown cohort perceived similar levels of well-being as those in the prepandemic cohort (*P*=.25), those in the intralockdown cohort perceived lower general well-being than those in the postlockdown cohort (*P*=.02). No differences in students’ general well-being were found between the postlockdown and prepandemic cohorts (*P*=.50).

#### Study-Related Emotional Well-being

MANOVA results for study-related stress and the 2 test anxiety subscales—agitation and worry—with student cohort as independent factor did not reveal any differences among the cohorts for the combined dependent variables (*V*=0.004; *F*_6,1566_=0.56; *P*=.76; *η*_p_^2^=0.002).

#### Academic Self-perception

MANOVA results for academic self-concept and study-related self-efficacy with student cohort as independent factor showed no cohort effect on the combined dependent variables (*V*=0.006; *F*_4,1568_=1.16; *P*=.33; *η*_p_^2^=0.003).

#### Motivation

MANOVA results for the 2 achievement motivation subscales—probability of success and probability of failure—as well as study-related flow with student cohort as independent factor revealed differences among the student cohorts (*V*=0.018; *F*_6,1566_=2.40; *P*=.03; *η*_p_^2^=0.009). Univariate analyses demonstrated a cohort effect on study-related flow (*F*_2,784_=4.90; *P*=.008; *η*_p_^2^=0.012) but neither on probability of success (*F*_2,784_=1.27; *P*=.28; *η*_p_^2^=0.003) nor on probability of failure (*F*_2,784_=2.65; *P*=.07; *η*_p_^2^=0.007).

As for students’ study-related flow, Bonferroni-corrected post hoc analyses indicated that students in the intralockdown cohort reported lower flow levels than those in the prepandemic cohort (*P*=.007). Furthermore, students in the intralockdown cohort reported less study-related flow than those in the postlockdown cohort (*P*=.04). No differences were found between the postlockdown and prepandemic cohorts (*P*=.50).

#### Academic Self-regulation

MANOVA results for students’ concentration, frequency of study activities, and procrastination with student cohort as independent factor showed a cohort effect on the combined dependent variables (*V*=0.022; *F*_6,1566_=2.94; *P*=.008; η_p_^2^=0.011). Univariate analyses revealed that students’ concentration differed among the cohorts (*F*_2,784_=6.61; *P*<.001; *η*_p_^2^=0.017). However, no differences were found for students’ frequency of study activities (*F*_2,784_=0.84; *P*=.43; *η*_p_^2^=0.002) or for students’ procrastination (*F*_2,784_=1.95; *P*=.14; η_p_^2^=0.005).

Bonferroni-corrected post hoc comparisons for students’ concentration showed that students in the intralockdown cohort had more difficulties concentrating on their study activities than students in the prepandemic cohort (*P*=.001). Students in the intralockdown cohort also reported lower concentration levels than those in the postlockdown cohort (*P*=.04). No difference in concentration was found between the postlockdown and prepandemic cohorts (*P*=.31).

## Discussion

### Principal Findings

This study is one of few comprehensive investigations into the effects of 2 of the COVID-19 lockdowns in Germany on students’ mental health incorporating a prepandemic control measure. Specifically, this study compared 3 student cohorts (a prepandemic, postlockdown, and intralockdown cohort) regarding various factors concerning students’ emotional well-being and academic functioning.

#### General Emotional Well-being

As for students’ general emotional well-being, including positive and negative affect, general well-being, and perceived stress, the results of our study indicated support for our hypotheses only for general well-being. Particularly, students assessed during the lockdown in Germany (ie, intralockdown cohort) experienced decreased general well-being (hypothesis 1c) compared with students assessed after lockdown-related restrictions were lifted (ie, postlockdown cohort). Furthermore, in line with our hypotheses, no differences regarding students’ well-being were observed between the postlockdown and prepandemic cohorts. These results are in accordance with existing empirical evidence showing that well-being–related detriments can emerge as a consequence of COVID-19 lockdown provisions but may be rapidly attenuated once restrictions are eased [7] or lifted [8]. However, it should be noted that all student cohorts reported concerningly low well-being scores, which were only exacerbated during the lockdown. These findings call attention to the general vulnerability of individuals in tertiary education [12,28-30] and the necessity of mental health programs particularly designed to help students during the COVID-19 crisis and beyond.

Contrary to our hypotheses, students’ positive (hypothesis 1a) and negative affect (hypothesis 1b) as well as stress (hypothesis 1d) did not differ among the 3 cohorts. However, for all cohorts, positive affect was only moderate, and stress presented itself as rather high, which appears to be complementary to the low well-being scores. It should be noted that, in the current literature, there is indication for an increase in negative affect and stress in students during the COVID-19 lockdowns [7,8,12]. However, there are various possible explanations for the absence of the expected differences regarding these outcomes in this research.

For instance, it may be that affect and stress were less affected by the lockdown-related restrictions as many students relocated to their caregivers’ place. Recent research has shown that relocating can reduce some of the experienced material and psychological burden during the pandemic and, thereby, attenuate perceived stress [27].

However, it is also possible that the measures used did not detect adverse changes that emerged among the student cohorts. As for positive and negative affect, it may be that, owing to low general well-being [64] and the social deprivation [44,65] precipitated by the COVID-19 lockdowns, students have experienced dampened emotionality. Thus, the mood measures used assessing high-activity emotional expressions (ie, anger, hostility, wakefulness, and determination) may have been inadequate to capture lockdown-related detriments. In other words, comprehensive measures encompassing low-activity emotions similar to those used in the applied well-being measure (eg, calm and relaxed, interested, and in good spirits) may allow for deeper insights into the lockdown effects on students’ mental health. Similarly, the stress measure used may not be an adequate indicator of lockdown-related detriments. A recent longitudinal study comparing students’ stress before and after the onset of the COVID-19 pandemic in Germany corroborates the absence of stress differences because of the lockdown [66]. However, the study revealed shifts in students’ behavior and experience patterns such as that healthy (ie, effective stress coping and positive study-related behaviors) and overexertion (ie, ineffective stress coping and study-related overcommitment) tendencies decreased, whereas unambitious (ie, effective stress coping and low study-related commitment) and burnout (ie, ineffective stress coping and low study-related ambition) patterns increased between the prepandemic and intrapandemic measure. Thus, although the COVID-19 lockdowns may not have affected reported stress, underlying stress perceptions and coping behaviors may have changed.

In line with this, it is also noteworthy that, unlike most existing studies [8,12-14], our study investigated well-being–related detriments both in the earlier phase (ie, the postlockdown cohort) and in a later phase (ie, the intralockdown cohort) of the pandemic. Consequently, students had more experience with lockdown-related restrictions, which may have led to the development of various adaptive and maladaptive coping strategies as well as behavior and experience patterns. With this, our results indicate that more research comparing different phases of the pandemic and different emotional expressions as well as coping patterns with regard to students’ general emotional well-being is needed. Previous research has started to investigate possible coping strategies used by students during the COVID-19 crisis [67-69]. However, this research is still extremely limited.

#### Academic Functioning

As for students’ academic functioning, our results showed support for some but not all outcome variables. Specifically, in line with our hypotheses, we found that study-related flow (hypothesis 2f) and concentration (hypothesis 2g) were lowest in the intralockdown cohort compared with the prepandemic and postlockdown cohorts.

These findings are in line with recent research demonstrating detriments to students’ motivation [14,31] and attention [39] associated with lockdown-precipitated social detriments and the shortcomings of remote learning formats. As for study-related flow in particular, the results obtained are in line with research suggesting adverse effects brought about by uncertainty regarding study and exam requirements, insufficient interstudent and student-teacher communication, and enhanced and often undefined workload [9]. Important components of study-related flow are clear definitions of study goals, frequent feedback, and high perceived control [60]. Consequently, the lack of such aspects may hinder study-related flow. These findings are not just of relevance as diminished study-related flow and concentration may directly impair students’ academic functioning but also as they are associated with the frequency of study activities. Thus, detriments in these academic areas may also worsen study engagement in general. It should be noted that, contrary to our hypotheses, we did not identify any differences in study-related flow and concentration between the postlockdown and prepandemic cohorts. This result indicates that despite potential problems arising in the context of remote learning, the remote learning format alone may not lead to academic detriments as long as it does not co-occur with other pandemic-related stressors such as social detriments. However, more research is needed to support this claim.

In contrast to our hypotheses, we did not identify any cohort effects on students’ study-related stress (hypothesis 2a), test anxiety (hypothesis 2b), academic self-concept (hypothesis 2c), self-efficacy (hypothesis 2d), achievement motivation (hypothesis 2e), frequency of study activities (hypothesis 2h), and procrastination (hypothesis 2i).

There are multiple possible explanations for the lack of observed differences. Most of these factors concerning academic functioning represent rather stable person characteristics; something that is also reflected in the measures used to assess the respective variables. It is possible that COVID-19 lockdown measures and remote learning—despite being incisive life events—simply do not unsettle stable person characteristics as easily as more variable aspects of academic functioning. Research also suggests that factors such as general self-efficacy may act as protective factors buffering the individual from potential COVID-19–related detriments [70]. Similarly, it is possible that adverse person characteristics such as the tendency to procrastinate act as risk factors enhancing the adverse consequences arising from COVID-19–related academic stressors. In fact, in our study, academic self-concept and study-related self-efficacy scores were rather high; however, students still experienced some general emotional well-being– and academic functioning–related impairments, albeit the effects were generally small. Consequently, more investigations into protective and risk factors are needed to identify potential implications for interventional programs on students’ academic functioning during the crisis.

Another explanation for the lack of differences concerning the aforementioned factors could be that some aspects of academic functioning related to the exam phase of the semester, which was still over a month away at the time students participated in the web-based survey. Consequently, such measures (ie, study-related stress, test anxiety, study-related self-efficacy, and achievement motivation) may have not been able to identify strains that would have occurred shortly before the exam phase.

Finally, it is important to note that, as part of a larger longitudinal study, this study only included individuals who were highly versed in the use of mobile technologies, particularly the smartphone. Earlier, we referred to prior work suggesting that, besides social detriments and remote learning in general, heightened screen time [34,36,37] may further engender academic impairments. However, students in our study possessed sound knowledge of their devices as—for study participation—they had to use their smartphones on a daily basis. Consequently, these students may have encountered few complications (eg, technical problems and internet access) with regard to remote learning formats. Moreover, as students participated in our study with the goal of reducing their smartphone interference while studying, it is highly probable that most of them had been exhibiting smartphone and technology overuse tendencies already before the pandemic. Thus, the potential academic detriments of heightened screen time because of COVID-19 lockdowns may have simply not become apparent in our student sample.

Still, in light of the revealed impairments to students’ concentration and study-related flow as well as the uncertainty regarding the development of the COVID-19 crisis, it appears reasonable to develop academic programs promoting students’ academic functioning. This is especially important as remote learning formats are likely to be continued in one way or another.

### Limitations

Despite its contributions, this study also has limitations. First, it must be noted that we investigated between-subject data; thus, students in the different cohorts were not the same individuals and, therefore, may differ in the general emotional well-being and academic functioning aspects that were assessed in this study. Moreover, even though we drew from a nationwide sample, some sociodemographic factors were not equally distributed across cohorts. Future comprehensive research with representative study samples incorporating multiple measurement points for one or several cohorts as has been done in few existing studies would be beneficial to better understand the effects of the COVID-19 crisis on the student population. Alternatively, it is possible that students who were particularly impaired because of COVID-19 lockdown measures and emergency remote learning discontinued their studies; thus, cohorts may not be easily comparable because of unknown student fluctuation. This possibility should be regarded in future research. Furthermore, we do not know how students felt during the first lockdown. Clearly, this insight would be particularly valuable to answer questions on whether coping has been, at least to some degree, successful over time and whether interventional programs are needed. In addition, our study can only provide information regarding the first 2 lockdowns in Germany. Thus, we want to emphasize the importance of continuing the investigation of the ongoing effects of the COVID-19 pandemic on students and the general population. We want to encourage future research to address these issues by analyzing available data from different time points during the pandemic with regard to students’ general emotional well-being and academic functioning.

### Conclusions

This study contributes to the COVID-19 literature regarding students’ mental health. It is one of few studies incorporating a prepandemic control measure and a measure during the later phase of the pandemic while also providing broad insights into various mental health aspects relating to students’ general well-being and academic functioning. In summary, this study showed that students experience both general emotional well-being–related detriments (ie, worsened general well-being) as well as some impairments to their academic functioning (ie, decreased concentration and study-related flow). Thus, in light of the ongoing crisis, possible interventional approaches implementable within educational institutions should be addressed.

## Appendix

Multimedia Appendix 1 Flow of participants.

Multimedia Appendix 2 Multivariate analysis of covariance results for the combined dependent variables positive affect, negative affect, general well-being, and perceived stress.

Multimedia Appendix 3 Multivariate analysis of covariance results for the combined dependent variables study-related stress, test anxiety (agitation), and test anxiety (worry).

Multimedia Appendix 4 Multivariate analysis of covariance results for the combined dependent variables academic self-concept and self-efficacy.

Multimedia Appendix 5 Multivariate analysis of covariance results for the combined dependent variables achievement motivation (probability of success), achievement motivation (probability of failure), and study-related flow.

Multimedia Appendix 6 Multivariate analysis of covariance results for the combined dependent variables concentration, frequency of study activities, and procrastination.

## Abbreviations

MANOVA: multivariate analysis of variance
