# Supplementary figures and images for: Students' Emotional Well-being and Academic Functioning Before, During, and After Lockdown in Germany: Cohort Study
Source: JMIR Form Res. 2022 Nov 15;6(11):e34388. doi: 10.2196/34388 (PMC9668332; doi:10.2196/34388)

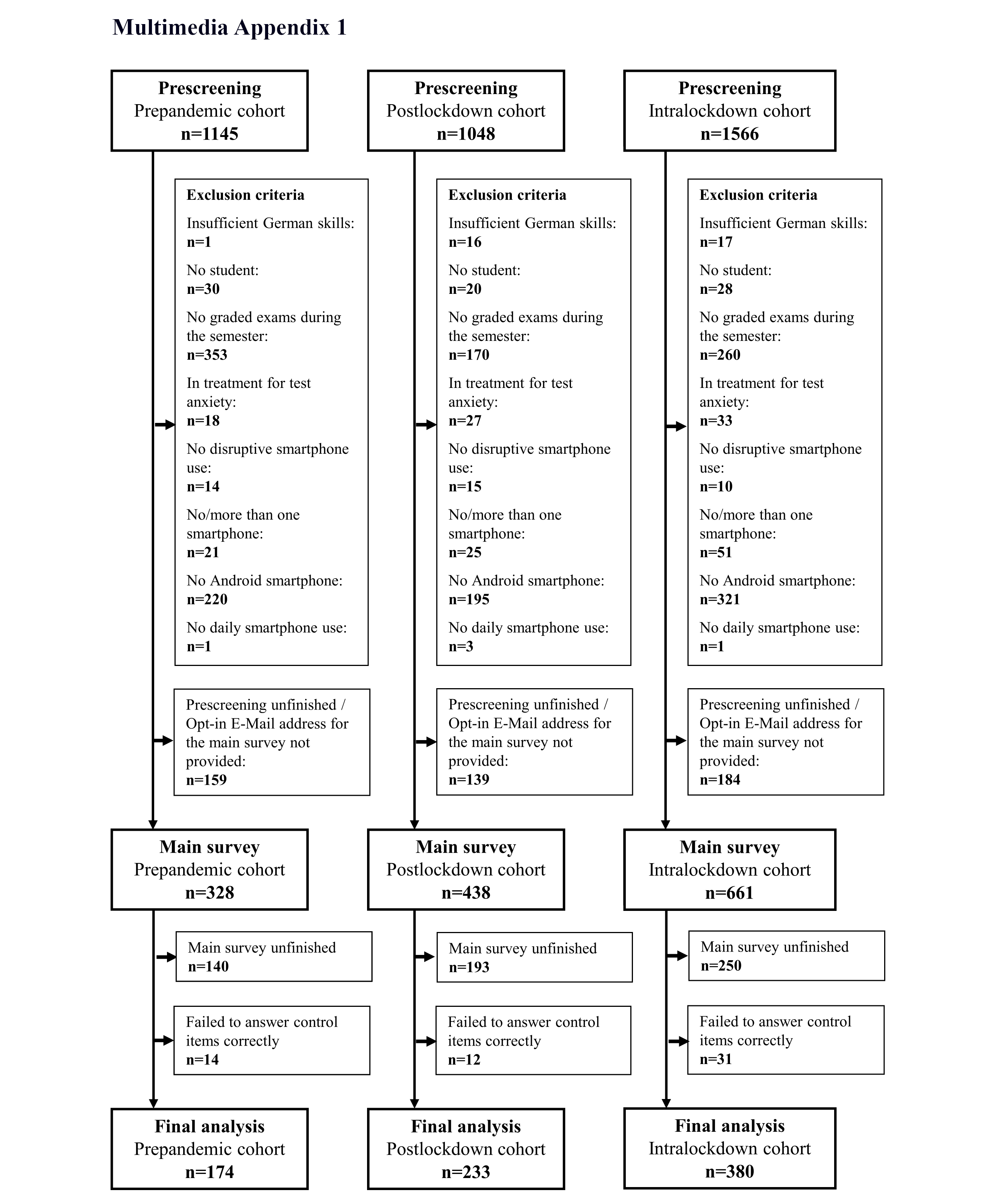

Supplement: Multimedia Appendix 1 [file formative_v6i11e34388_app1.png]
