# Supplementary material for: Students' Emotional Well-being and Academic Functioning Before, During, and After Lockdown in Germany: Cohort Study
Source: JMIR Form Res. 2022 Nov 15;6(11):e34388. doi: 10.2196/34388 (PMC9668332; doi:10.2196/34388)
Supplement: Multimedia Appendix 2 [file formative_v6i11e34388_app2.pdf]

Multimedia Appendix 2

Table S1. Multivariate analysis of covariance results for the combined dependent variables positive affect, negative affect, general well-being, and perceived stress.

Multivariate analysis					Univariate comparisons											
**Covariates**					**Positive affect**			**Negative affect**			**General well-being**			**Perceived stress**		
	V	*F(4, 776)*	*P*	η_p^2	*F*(1, 779)	*P*	η_p^2	*F*(1, 779)	*P*	η_p^2	*F*(1, 779)	*P*	η_p^2	*F*(1, 779)	*P*	η_p^2
Gender	0.007	1.37	.24	.007	0.054	.82	<.001	.270	.60	<.001	2.88	.09	.004	1.99	.16	.003
Age	0.006	1.095	.36	.006	1.13	.29	.001	2.12	.15	.003	3.12	.08	.004	0.63	.43	.001
Semester	0.002	0.40	.81	.002	0.32	.57	.001	0.17	.68	<.001	0.078	.78	<.001	0.021	.88	<.001
Exam count	0.003	0.50	.73	.003	0.21	.65	<.001	1.06	.30	.001	0.47	.49	.001	0.025	.88	<.001
**Fixed factors**																
	V	*F(8,1554)*	*P*	η_p^2	*F*(2,779)	*P*	η_p^2	*F*(2,779)	*P*	η_p^2	*F*(2,779)	*P*	η_p^2	*F*(2,779)	*P*	η_p^2
Cohort	0.020	1.96	.048	.010	3.04	.049	.008	0.90	.41	.002	3.56	.03	.009	2.79	.06	.007
