# Supplementary material for: Students' Emotional Well-being and Academic Functioning Before, During, and After Lockdown in Germany: Cohort Study
Source: JMIR Form Res. 2022 Nov 15;6(11):e34388. doi: 10.2196/34388 (PMC9668332; doi:10.2196/34388)
Supplement: Multimedia Appendix 3 [file formative_v6i11e34388_app3.pdf]

Multimedia Appendix 3

Table S2. Multivariate analysis of covariance results for the combined dependent variables study-related stress, test anxiety (agitation), and test anxiety (worry).

Multivariate analysis					Univariate comparisons								
**Covariates**					**Study-related stress**			**Test anxiety (agitation)**			**Test anxiety (worry)**		
	V	*F(3, 778)*	*P*	η_p^2	*F*(1, 780)	*P*	η_p^2	*F*(1, 780)	*P*	η_p^2	*F*(1, 780)	*P*	η_p^2
Gender	0.008	2.15	.09	.008	1.00	.32	.001	3.68	.06	.005	5.59	.02	.007
Age	0.004	1.12	.34	.004	2.49	.12	.003	2.00	.16	.003	0.29	.59	<.001
Semester	0.004	0.98	.40	.004	0.40	.53	.001	0.042	.84	<.001	0.81	.37	.001
Exam count	0.029	7.66	<.001	.029	6.40	.01	.008	1.69	.19	.002	0.46	.50	.001
**Fixed factors**													
	V	*F(6,1558)*	*P*	η_p^2	*F*(2,780)	*P*	η_p^2	*F*(2,780)	*P*	η_p^2	*F*(2,780)	*P*	η_p^2
Cohort	0.004	0.54	.08	.002	0.074	.93	<.001	0.67	.51	.002	0.15	.86	<.001
