# Supplementary material for: Students' Emotional Well-being and Academic Functioning Before, During, and After Lockdown in Germany: Cohort Study
Source: JMIR Form Res. 2022 Nov 15;6(11):e34388. doi: 10.2196/34388 (PMC9668332; doi:10.2196/34388)
Supplement: Multimedia Appendix 4 [file formative_v6i11e34388_app4.pdf]

Multimedia Appendix 4

Table S3. Multivariate analysis of covariance results for the combined dependent variables academic self-concept and self-efficacy.

Multivariate analysis					Univariate comparisons					
**Covariates**					**Academic self-concept**			**Self-efficacy**		
	V	*F(2, 778)*	*P*	η_p^2	*F*(1, 780)	*P*	η_p^2	*F*(1, 780)	*P*	η_p^2
Gender	0.002	0.60	.55	.002	0.61	.44	.001	1.18	.28	.002
Age	0.006	2.20	.11	.006	4.10	.04	.005	0.71	.40	.001
Semester	0.006	2.24	.11	.006	3.79	.05	.005	3.52	.06	.004
Exam count	0.08	2.97	.05	.008	5.93	.02	.008	2.08	.15	.003
**Fixed factors**										
	V	*F(4,1560)*	*P*	η_p^2	*F*(2,780)	*P*	η_p^2	*F*(2,780)	*P*	η_p^2
Cohort	0.005	0.93	.45	.002	1.72	.18	.004	1.01	.36	.003
