# Supplementary material for: Students' Emotional Well-being and Academic Functioning Before, During, and After Lockdown in Germany: Cohort Study
Source: JMIR Form Res. 2022 Nov 15;6(11):e34388. doi: 10.2196/34388 (PMC9668332; doi:10.2196/34388)
Supplement: Multimedia Appendix 5 [file formative_v6i11e34388_app5.pdf]

Multimedia Appendix 5

Table S4. Multivariate analysis of covariance results for the combined dependent variables achievement motivation (probability of success), achievement motivation (probability of failure), and study-related flow.

Multivariate analysis					Univariate comparisons								
**Covariates**					**Motivation (Success)**			**Motivation (Failure)**			**Study-related flow**		
	V	*F(3, 778)*	*P*	η_p^2	*F*(1, 780)	*P*	η_p^2	*F*(1, 780)	*P*	η_p^2	*F*(1, 780)	*P*	η_p^2
Gender	0.006	1.51	.21	.006	0.070	.79	<.001	4.14	.04	.005	0.040	.08	<.001
Age	0.001	0.25	.86	.001	0.34	.56	<.001	0.11	.74	<.001	0.002	.97	<.001
Semester	0.015	3.92	.009	.015	2.11	.15	.003	6.99	.008	.009	1.17	.28	.001
Exam count	0.017	4.55	.004	.017	6.29	.01	.008	0.011	.92	<.001	2.28	.13	.003
**Fixed factors**													
	V	*F(6,1558)*	*P*	η_p^2	*F*(2,780)	*P*	η_p^2	*F*(2,780)	*P*	η_p^2	*F*(2,780)	*P*	η_p^2
Cohort	0.017	2.21	.04	.008	0.83	.44	.002	1.38	.25	.004	5.45	.004	.014
