# Supplementary material for: Students' Emotional Well-being and Academic Functioning Before, During, and After Lockdown in Germany: Cohort Study
Source: JMIR Form Res. 2022 Nov 15;6(11):e34388. doi: 10.2196/34388 (PMC9668332; doi:10.2196/34388)
Supplement: Multimedia Appendix 6 [file formative_v6i11e34388_app6.pdf]

Multimedia Appendix 6

Table S5. Multivariate analysis of covariance results for the combined dependent variables concentration, frequency of study activities, and procrastination.

Multivariate analysis					Univariate comparisons								
**Covariates**					**Concentration**			**Frequency of study activities**			**Procrastination**		
	V	*F(3, 778)*	*P*	η_p^2	*F*(1, 780)	*P*	η_p^2	*F*(1, 780)	*P*	η_p^2	*F*(1, 780)	*P*	η_p^2
Gender	0.013	3.33	.02	.013	4.06	.04	.005	0.20	.65	<.001	1.57	.21	.002
Age	0.011	2.87	.04	.011	0.11	.74	<.001	0.32	.57	<.001	4.83	.03	.006
Semester	0.003	0.80	.05	.003	0.18	.68	<.001	1.55	.21	.002	0.50	.48	.001
Exam count	0.025	6.78	<.001	.025	2.96	.09	.004	17.31	<.001	.022	0.27	.60	<.001
**Fixed factors**													
	V	*F(6,1558)*	*P*	η_p^2	*F*(2,780)	*P*	η_p^2	*F*(2,780)	*P*	η_p^2	*F*(2,780)	*P*	η_p^2
Cohort	0.023	2.98	.007	.011	7.07	.001	.018	0.47	.63	.001	1.83	.16	.005
